# Infant Injury Prevention Education for Pregnant Women Attending Antenatal Class: A Quasi-Experimental Study

**DOI:** 10.3390/ijerph18179393

**Published:** 2021-09-06

**Authors:** Chikako Honda, Takashi Naruse, Hayato Yamana, Noriko Yamamoto-Mitani

**Affiliations:** 1 Department of Community Health Nursing, Division of Health Sciences and Nursing, Graduate School of Medicine, The University of Tokyo, Tokyo 113-0033, Japan; noriko-tky@g.ecc.u-tokyo.ac.jp; 2 Global Nursing Research Center, Graduate School of Medicine, The University of Tokyo, Tokyo 113-0033, Japan; takanaruse@g.ecc.u-tokyo.ac.jp; 3 Department of Health Services Research, Graduate School of Medicine, The University of Tokyo, Tokyo 113-0033, Japan; yamana@m.u-tokyo.ac.jp

**Keywords:** infant care practices, injury prevention, pregnancy, program evaluation, safety practice, behavior change

## Abstract

It is important to educate caregivers in order to prevent infant injuries. However, there have been few studies on the effects of education on pregnant women. This study aimed to evaluate the effects of injury prevention group education on this group. Study participants were recruited from a group of pregnant mothers attending an antenatal class in Tokyo. Participants were assigned to either the intervention or control group based on the month in which they attended the existing antenatal class. Both groups received a leaflet on injury prevention, but only the intervention group received an additional short one-shot lecture. The implementation of each of the nine safety practices was assessed during home visits after childbirth. Of the 131 study participants (56 in the control group and 75 in the intervention group), 106 (80.9%) received home visits after birth. Mothers in the intervention group implemented three practices significantly more than those in the control group: Keep soft objects away from the baby’s head (38.3% vs. 13.0%), Do not place your baby on a high surface (74.6% vs. 52.2%), and Use the baby carrier correctly (93.3% vs. 76.1%). In the future, we plan to follow up the participants to evaluate the program’s long-term effects, and to continue to improve the program.

## 1. Introduction

Unintentional injury is a major cause of death and morbidity in children, and injury prevention is a global public health challenge that requires a strategic response [1]. Of unintended injuries among infants (<12 months old), 88.6% occurred at home [2]. Immediately after birth, accidents such as falls and suffocation often occur, leading to fatal or non-fatal injuries [3,4,5].

One-fifth of the first hospital visits due to injury, accidental ingestion, or burns within the first 12 months of life occurred within the first four months [6]. In Japan, injury prevention education for parents is mainly provided after four months of age, and there are few opportunities for injury prevention education before that time [7].

We focused on pregnancy as the best time for early education. Pregnant mothers want to receive information on injury prevention [8]. Previous studies have shown that home visits starting from pregnancy improved parental injury prevention [9,10,11] and decreased the incidence of injuries [12]. The previous four studies adopted individual intervention [9,10,11,12], but this was resource-consuming and had modest impact [13]. We focused on group education as a less costly and more feasible way to provide information [14,15].

In Japan, almost all pregnant women register their pregnancies with a municipality, and most municipalities provide information to pregnant women by providing antenatal classes [16]. In Tokyo, 52.8% of individuals who register their pregnancies attend antenatal classes for primipara [17]. Providing injury prevention education to pregnant women in existing antenatal classes in municipalities can reach many pregnant women without additional effort.

This study aimed to evaluate the implementation of safety practices (SPs) after childbirth by providing an infant injury prevention program for pregnant women. We hypothesized that pregnant women who received group education about infant injury prevention would enhance their intention to implement SPs and increase SPs after birth. In the Theory of Planned Behavior (TPB) and the Theory of Reasoned Action (TRA), behavioral intention is the most significant factor that defines personal behavior [18].

## 2. Methods

### 2.1. Study Design and Setting

This quasi-experimental study (nonequivalent control group design) was conducted in antenatal classes at a public health center in city X of the Tokyo metropolitan area, Japan, from November 2017 to June 2018. The number of pregnancies reported in that city was 2594 in 2017. The number of participants in the antenatal classes was 1461 (56.3% of all registered pregnancies, including those with a second or later child in city X [17]. The antenatal class consisted of three sessions over three weeks. The first session included the birth story and bathing practice, the second session included dental information and nutrition during pregnancy, and the third session included postnatal social resources and interactions with senior mothers and children.

We set up an expert panel consisting of 13 members (three Public Health Nurses (PHNs), three Midwives (MWs), a pediatric emergency physician, a mother and a father practicing health education for parents, an injury prevention researcher, and three public health nursing researchers). We collaborated on the entire study, including program development, while checking its validity.

### 2.2. Eligibility and Enrollment

The inclusion criteria for our study were: a pregnant woman expecting her first child who was in a stable period of pregnancy and who could understand Japanese. The women attending the antenatal class at public health center in city X had to be communicable with in Japanese, within a stable period (approximately 24–36 weeks’ gestation), and have had their first pregnancy. Thus, all the participants met our inclusion criteria and were recruited as subjects of this study.

At the first antenatal class, the first author gave oral presentations about the study to all attendees. After that, we handed over the informed consent forms and a baseline questionnaire to those who wished to participate. Answers and written consent were collected after the antenatal class.

The assignment was as follows: pregnant women who attended the antenatal class in the first four months during the study period were assigned to the control group (November 2017 to February 2018), and those who attended the latter four months were assigned to the intervention group (March to June 2018). This assignment was adopted to avoid operational complexity and to reduce contamination when there was sharing of knowledge in the community due to the interaction of both groups’ attendees, because this study used the existing antenatal classes in the community.

### 2.3. Intervention

For attendees in the intervention group, a one-shot educational program was provided after the second session. For all attendees in both groups, the existing leaflet was given in the first session, and they were encouraged to read it at home. The leaflet had been published by the Tokyo Metropolitan Government and covered general topics related to infant injury prevention [19].

#### 2.3.1. Educational Program

The educational program was developed using the following steps.

##### Identifying Target Injuries and SP Recommendation

First, based on the national prevalence of child injuries [20,21], we identified four types of injury that our intervention should contribute to preventing: suffocation, falls, accidental ingestion, and burns. Next, the SPs that our intervention aimed to promote were identified. The first author extracted SPs from domestic and international literature, discussed them with six experts (a pediatrician, a pediatric emergency physician, a medical examiner, an injury-prevention researcher, PHN, and MW), and finally determined nine SPs as target SPs for the above four injuries after validation by the expert panel (Table 1).

Hereafter, we use the shortened phrase as follows:

(1) Use a firm mattress
(2) Keep soft objects away from the baby’s head
(3) Make sure that there is no space
(4) Do not use a bed guard
(5) Do not place your baby on a high surface
(6) Keep crib sides raised
(7) Use the baby carrier correctly
(8) Do not place small objects
(9) Keep something hot away from the baby

##### Development of the Program Content

First, we interviewed 15 PHNs with experience in planning and managing injury prevention education and four pregnant women expecting their first child, in order to determine a logic model for the program. By involving program implementers and users from the early stages of program development, we focused on visualizing the program, sharing goals, and making the program highly feasible [22]. An expert panel verified the logic model created.

The authors followed the logic model and developed a script for lectures that reflected the content of each intervention session. The contents of the educational program are shown in Table 2. The program was structured according to the three situations of living with a newborn: “Safety in putting baby to sleep”, “Safety in carrying baby”, and “Safety in room”. These situations included knowledge about nine SPs. As an introduction to the program, a video was seen (about two minutes long), showing how babies could move their arms and legs, even when they were not expected to move, until they could turn over in bed, along with a case of an accident that occurred close to home involving a baby up to four months of age. We also used the results of an earlier survey of pregnant women’s knowledge about SPs and their intention to implement SPs [23] as a reference when developing the program content. The panel of experts agreed upon the script and content. We also created a handout.

### 2.4. Outcomes

The primary outcome was the implementation status of each of the nine SPs. They were measured during home visits after giving birth (T3) by MWs or PHNs. Postpartum home visits are conducted for all newborns in Japan, with an implementation rate of 99.7% in city X. In this study, as soon as the public health center received the “birth notification” from the study participants, a trained PHN or MW visited the home and checked the status of SP implementation by using the evaluation form. The evaluation form and its contents were developed by the panel of experts.

SP (1), (2), (3), (4), (7), and (8) were checked by direct observation to see if the participant has “implemented” or “not implemented” the SPs. SP (5), (6), and (9) were checked by asking the participant questions and receiving responses on a five-point scale from “Not at all” to “Always implemented”. The status of “Always implemented” was rated as “Implemented”. We also obtained other postnatal information through the public health center’s maternal and child health system and by the self-questionnaire that MWs or PHNs handed out during home visits. These were all managed by ID, and the information was pieced together.

The secondary outcomes were the intention to implement SPs and knowledge of SPs. SPs’ intention and knowledge were assessed by self-report using a questionnaire completed at the first session in the antenatal class (T1) and at 36 weeks gestation (T2). Each intention regarding the nine SPs was asked about with five Likert-scale responses from “I will implement it necessarily” to “I would not implement it”; the response “I will implement it necessarily” was rated as “With intention.” The participants were asked about the nine SPs via two to four questions on each SP using a five-point Likert scale, with responses ranging from “Absolutely right” to “Absolutely wrong”; the responses “Absolutely right” and “Right” were rated as “Correct answer”. We developed questions on intention and knowledge with the input of pediatric emergency physicians and injury prevention researchers. 

To complete the questionnaires, participants received a 1500-yen (approximately USD 13) gift card.

### 2.5. Sample Size

We assumed a mean of 60% implementation for SP [24] and estimated the difference between the two groups after the intervention, which was 0.8 [13,25]. Using an effect size of 0.5, α of 0.05, and β of 0.20, the sample size was 128 participants. We set the goal of recruiting a total of 153 participants, considering dropout.

### 2.6. Statistical Analysis

Assignment to the intervention or control group was based on allocation at T1. The primary and secondary outcomes were analyzed in participants who received home visits at T3 and those who replied to the mail survey at T2.

The evaluation of the intervention effects was conducted as follows. For the main outcome, the number and proportion of people who implemented each SP were calculated. Fisher’s exact test was used to compare the proportion of persons who implemented the SPs between the intervention and control groups. For the second outcome, the number and proportion of people who intended to implement each SP were calculated, and the proportion of persons showing intention between the two groups was compared using Fisher’s exact test. The mean and standard deviation of the number of correct knowledge answers for each SP were also calculated and compared using the Mann-Whitney U test.

All statistical analyses were performed using the Statistical Package for Social Science version 24.0 software (IBM, Armonk, NY, USA). All P-values were two-sided, and a P-value of less than 0.05 was considered statistically significant.

### 2.7. Ethical Considerations

This study was conducted in accordance with the Declaration of Helsinki and was approved by the ethics review board of the University of Tokyo (IRB file No.11748) on 11 October 2017.

## 3. Results

### 3.1. Baseline Characteristics of Study Participants

**Figure 1:**
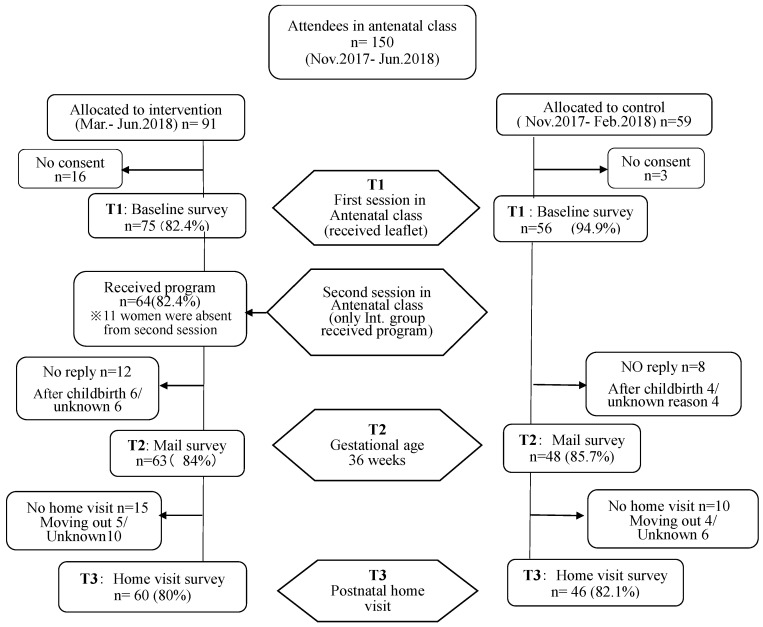
Flowchart of participant registration and progress.

The participants’ entry and flow through the study are shown in Figure 1.

Of the 150 pregnant women who attended the first antenatal session from November 2017 to June 2018, 131 (87.3%) agreed to participate in the study. The intervention group had 75 participants, while the control group had 56 participants.

Table 3 shows the demographic characteristics of the respondents stratified by the intervention and control groups. The mean age of participants was 33.3 years, and the gestational age was 28.7 weeks. More than half had some college or more education (60.3%) and were not living with their grandparents (92.4 %). Of the households, 59.5% had an annual income of more than seven million yen. There were no statistically significant demographic differences between the two groups.

### 3.2. Status of Implementation of Safety Practices after Giving Birth

A total of 106 home visits (intervention group: 60; control group: 46) were implemented (response rate: 80.9 %). The average timing at which the visits were conducted was 51.6 days postpartum. There was no significant difference in the rate of visitation or demographic differences between the two groups.

Table 4 shows the rates of the nine SP implementations for the intervention and control groups at T3.

The rates of implementation of each SP showed that the rate of implementation of the following three SPs was significantly higher in the intervention group than in the control group: (2) Keep soft objects away from the baby’s head (*p* = 0.004); (5) Do not place your baby on a high surface (*p* = 0.023); and (7) Use the baby carrier correctly (*p* = 0.022). For three items, namely, (3), (4), and (6), there was no difference between the intervention and control groups in the proportion of respondents who did not own a baby crib, had never used a baby crib even when out and about, or did not own an adult bed.

### 3.3. Status of Intention and Knowledge of Safety Practices at Baseline and 36 Weeks Gestational Age

Table 5 shows the status of participants’ intention to implement SPs and knowledge of SPs at the baseline (T1) and 36 weeks gestation (T2).

At T1, five of the nine items showed more than 80% of participants’ intention to implement ((5), (6), (7), (8), (9)). However, only 30%–60% of participants intended to implement the four SP items of asphyxiation prevention ((1), (2), (3), (4)). At T2, the rates of the intention to implement the following two SPs were significantly higher in the intervention group than in the control group: (3) Make sure that there is no space (*p* = 0.009), and (4) Do not use a bed guard (*p* < 0.001).

There was no difference between the intervention and control groups at T1 regarding the amount of correct knowledge. At T2, the number of correct answers in the intervention group for following the five SPs (2), (3), (4), (7), (8) was significantly higher than in the control group.

## 4. Discussion

The women in the intervention group implemented three SPs significantly more after childbirth than those in the control group. The results showed that group education for pregnant women was effective in increasing the implementation of three SPs. However, six of the SPs showed no difference in implementation rate. The reasons for this are discussed based on the results of the intention and behavior.

### 4.1. Effectiveness of the Intervention Program

In three SPs, namely, (2) Keep soft objects away from the baby’s head, (5) Do not place your baby on a high surface, and (7) Use the baby carrier correctly, participants in the intervention group implemented significantly more SPs than the control group. In these three SPs, even though there was no difference in each SP’s intentions rates at T2 between the intervention and control groups, the after-delivery implementation rates for each of the three SPs were significantly higher in the intervention than in the control group. The program could have influenced participants to act directly, even without their intentions.

The similarity of the three SPs were that they were behaviors that mothers themselves could easily implement without being influenced by other factors.

The Theory of Planned Behavior (TPB) defines behavioral intention as the most important determinant, and “perceived behavioral control” is one of the factors that define intention [18,26]. A meta-analysis of TPB found that there were routes where “perceived behavioral control” directly influenced behavior without intention [26,27]. “Perceived behavioral control” consists of the beliefs that one has the skills necessary to act (control beliefs) and that one can act as one wishes against barriers (perceived power). The mothers’ “perceived behavioral control” might have been enhanced by knowing the risks and correct behaviors via the program, which directly affected their behavior.

In the program, for (5), we corrected mothers’ perceptions (attitudes) such as “putting her to sleep on the floor is cold and pitiful,” and also showed them alternatives when there were specific barriers. In (2), we showed, using videos and photographs, the risk of suffocation caused by placing childcare items around the baby’s head. In (7), a demonstration showed how dolls fall due to improperly wearing a carrying strap and bad posture.

On the other hand, there was no difference in the rates of implementation of each SP between the intervention group and the control group in the following behaviors: (1) Use a firm mattress, (3) Make sure that there is no space, (4) Do not use a bed guard, (6) Keep crib sides raised, (8) Do not place small objects, and (9) Keep something hot away from the baby. In SP (1), (6), (8), and (9), there was no difference in the intention at T2 between the two groups. In other words, although SP (1, 6, 8, 9) and SP (2, 5, 7) had the same results in T2, the results differed in T3. This may be because factors other than mothers’ will influence SP behavior (1,6,8,9), and the mothers’ “perceived behavioral control” was less likely to influence behavior directly.

In a previous study, the following factors were associated with Japanese mothers’ car seat installation behaviors: child resistance to installation, annoyance in dealing with child resistance, and the low subjective norm of the husband [28]. Thus, factors other than the mother herself had a strong influence on this behavior. For example, if grandparents gave parents soft bedding, if the crib had a complicated structure to lift the guard, if the husband did not pay attention to the storage of daily necessities, or if a mother held her crying baby while cooking, SP (1), (6), (8), and (9) would not be implemented.

In SP (3) and (4), more mothers from the intervention groups intended to implement SP in T2. Since SP (3) and (4) were the items with the lowest rates of intention at the baseline (30.7% and 25.3%, respectively), it may have been easier to raise their intentions. Since (3) and (4) were behaviors that were easily influenced by factors other than the mother herself, they might not have led to the behavior’s implementation despite the mother’s intentions. For example, if parents had been presented with a bed guard, they might use it, or if parents bought a new mattress for the crib that was given to them, it might not fit. A low or absent sense of “perceived behavioral control” inhibits intention–behavior consistency [27].

Thus, the SP items for which the program did not promote behavioral implementation were SPs susceptible to influence by factors other than the mother herself.

Our first recommendation is to provide education for family members. Involving family members may facilitate the SP implementation influenced by factors external to mothers. Currently, during the COVID-19 pandemic, opportunities for face-to-face antenatal classes are decreasing. However, injury prevention programs using mobile technology [29] will be suitable to prevent infection. Complementing existing resources with new systems might enhance participation by fathers and grandparents who have difficulty attending face-to-face classes.

Second, we need to think about strategies for SPs (2) and (8), which had low implementation rates (31.7% and 38.3%, respectively). Although more than 90% of participants formed intentions after the intervention, their intentions did not lead to actions. SP (2) and (8) required the daily management of placement of frequently used items. Behaviors that require habitual management suppress the consistency of intention and behavior [27,30,31]. A study has suggested that education be incorporated into individuals’ lives and life transitions to promote highly habitual injury-prevention behavior [32]. However, intervention during pregnancy, a significant transition period, was not enough to promote the behaviors. Repeated post-partum education may be necessary based on each child’s stage of development.

This study showed that a short one-shot group education program during pregnancy could encourage several SPs by mothers without intensive interventions such as multiple home visits. Previous studies on injury prevention have focused on individual home visits or interventions at clinics or emergency department settings [9,10,11,12,29,33]. However, injury can happen to any child at any time. Therefore, we believe that group education for injury prevention in the community setting could serve as an important option to provide everyone with a wide range of minimum knowledge.

### 4.2. Limitations

This study has several limitations. First, the study participants’ mean age, education, and household income were generally higher than those of the national data [34,35,36]. The WHO and CDC cite younger mothers, shorter education, and lower income as risk factors for child injury. This study’s participants had the opposite predisposition to injury risk factors and might be more likely to take preventive action. The generalizability of our findings is limited. Second, because this was not a randomized controlled trial, unmeasured bias may have influenced the results. For example, seasonal differences may have affected the results. Although it is unlikely that the indoor environment changes significantly depending on the season due to the city center’s climate and the housing conditions in the city, the possibility of seasonal effects cannot be denied entirely. Third, although most of the primary outcome was measured using observation by a third party, the secondary outcome was self-reported using questionnaires. These outcomes might have been affected by desirability bias.

## 5. Conclusions

Significantly more pregnant women who attended group education on preventing infant injury from birth to four months of age implemented three SPs after birth: (2) Keep soft objects away from the baby’s head, (5) Do not place your baby on a high surface, and (7) Use the baby carrier correctly. In the future, we will follow up with the participants to evaluate the program’s long-term effects and to continue to improve the program based on these results.

## Figures and Tables

**Table 1:** Target injury and Safety Practices (SPs).

Injury	Safety Practice [SP]
Suffocation	SP (1) Use a firm mattress or futon
	SP (2) Keep soft objects away from the baby’s head in the baby’s sleep area
	SP (3) Make sure that there is no space between the mattress and bed frame
	SP (4) Do not use a bed guard until 18 months
Falls	SP (5) Do not place your baby on high surfaces, such as sofas or tables
	SP (6) Keep crib sides raised whenever you leave your baby in the crib
	SP (7) Drop your waist and support baby’s head with one hand when you lean forward while using a baby carrier.
Accidental ingestion	SP (8) Keep medicines, batteries, and small products in locked cabinets, or more than one meter above the floor
Burns	SP (9) Do not drink or carry hot liquids when holding your child

**Table 2:** Overview of educational program in the intervention group.

Section	Target SP	Aim	Measure	Time
Introduction		Aware of the importance of changing their lives to have a “safety perspective”	Imagine Watch short movie	4.5 min
Safety in putting baby to sleep	(1)(2)(3)(4)	Realize the necessity of each SP and the way of implementing SP	Demonstration Touch [mattress sample]	5 min
	(5)(6)		Watch picture	1.5 min
Safety in carrying baby	(7)(9)		Demonstration	3 min
Safety in room	(8)		Show real products [medicine, lithium button cell, etc.]	2 min
Summary		Realize that parents can be proactive in ensuring the safety of their baby/Believe that it is up to parents to keep their baby safe	Message by PPT	1.5 min

**Table 3:** Baseline characteristics of study participants (*n* = 131).

		Total (*n* = 131)		Intervention (*n* = 75)		Control (*n* = 56)		
		*n*	(%)	*n*	(%)	*n*	(%)	
		mean	(SD)	mean	(SD)	mean	(SD)	*p*
Age	(Range: 22–45)	33.3	(4.7)	33.3	(5.0)	33.2	(4.4)	0.869 ^a^
Gestational age	(Range: 21–38)	28.7	(3.9)	28.6	(4.2)	28.9	(3.5)	0.661 ^a^
Education level								
	Middle/High school	17	(13.0)	13	(17.6)	4	(7.1)	0.292 ^b^
	Junior college	34	(26.0)	20	(27.0)	14	(25.0)	
	College	68	(51.9)	36	(48.6)	32	(57.1)	
	Graduate school	11	(8.4)	5	(6.8)	6	(10.7)	
Employment status		39	(29.8)	26	(34.7)	13	(23.2)	0.204 ^b^
	Unemployed							
Partner’s age	(Range: 22–53)	35.0	(5.6)	35.1	(5.5)	34.9	(5.9)	0.846 ^a^
Partner’s education level								
	Middle/High school	10	(7.7)	6	(8.2)	4	(7.1)	0.864 ^b^
	Junior college	20	(15.3)	12	(16.4)	8	(14.3)	
	College	83	(63.4)	45	(61.6)	38	(67.9)	
	Graduate school	16	(12.2)	10	(13.7)	6	(10.7)	
Household annual income (thousands of yen)	0–3000	7	(5.3)	5	(6.8)	2	(3.6)	0.320 ^b^
	3000–4999	33	(25.2)	18	(24.3)	15	(26.8)	
	5000–6999	12	(9.2)	10	(13.5)	2	(3.6)	
	7000–9999	38	(29.0)	20	(27.0)	18	(32.1)	
	≥10,000	40	(30.5)	21	(28.4)	19	(33.9)	
Living with partner	No	6	(4.6)	5	(6.7)	1	(1.8)	0.238 ^c^
Living with grandparents	Yes	10	(7.6)	8	(10.7)	2	(3.6)	0.187 ^c^

^a^*t*-test, ^b^ chi-squared test, ^c^ Fisher’s exact test, Abbreviations: SD, Standard Deviation.

**Table 4:** Implementation status of participants’ safety practices at T3 (postnatal) (*n* = 106).

Safety Practice (SP)	Total (*n* = 106)		Intervention (*n* = 60)		Control (*n* = 46)		*p* ^a^
	*n*	(%)	*n*	(%)	*n*	(%)	
(1) Use a firm mattress	91	(85.8)	54	(90.0)	37	(80.4)	0.174
(2) Keep soft objects away from the baby’s head	29	(27.4)	23	(38.3)	6	(13.0)	0.004
(3) Make sure that there is no space *	40	(75.5)	25	(78.1)	15	(71.4)	0.746
(4) Do not use bed guard *	59	(93.7)	31	(91.2)	28	(96.6)	0.618
(5) Do not place your baby on high surfaces	68	(64.8)	44	(74.6)	24	(52.2)	0.023
(6) Keep crib sides raised *	34	(53.1)	22	(59.5)	12	(44.4	0.312
(7) Use the baby carrier correctly	91	(85.8)	56	(93.3)	35	(76.1)	0.022
(8) Do not place small objects	29	(27.4)	19	(31.7)	10	(21.7)	0.280
(9) Keep something hot away from the baby	90	(84.9)	68	(90.7)	36	(78.3)	0.108

^a^ Fisher’s exact test. * Respondents to (3) were only those who owned a crib, respondents to (4) were only those who owned an adult bed, and respondents to (6) were only those who had ever used a crib.

**Table 5:** Status of participants’ intention to implement safety practices and knowledge of safety practices at T1 (baseline) and T2 (*n* = 131/111).

	T1							T2						
	Total (*n* = 131)		Intervention (*n* = 75)		Control (*n* = 56)			Total (*n* = 111)		Intervention (*n* = 63)		Control (*n* = 48)		
	*n*	(%)	(%)	*n*	*n*	(%)	*p* ^c^	*n*	(%)	*n*	(%)	*n*	(%)	*p* ^c^
Intention to implement safety practices (SPs)														
(1) Use a firm mattress	82	(62.6)	47	(63.5)	35	(62.5)	1.00	105	(95.5)	61	(98.4)	44	(91.7)	0.165
(2) Keep soft objects away from the baby’s head	79	(60.3)	46	(63.9)	33	(58.9)	0.587	104	(93.7)	59	(93.7)	45	(93.8)	1.000
(3) Make sure that there is no space	44	(33.6)	23	(30.7)	21	(38.9)	0.352	73	(65.8)	48	(76.2)	25	(52.1)	0.009
(4) Do not use bed guard	33	(25.2)	19	(25.3)	14	(25.0)	1.00	34	(30.6)	29	(46.0)	5	(10.4)	<0.001
(5) Do not place your baby on high surfaces	104	(79.4)	61	(81.3)	43	(76.8)	0.663	101	(91.0)	59	(93.7)	42	(87.5)	0.324
(6) Keep crib sides raised	104	(79.4)	60	(81.1)	44	(78.6)	0.826	93	(83.8)	55	(87.3)	38	(79.2)	0.303
(7) Use the baby carrier correctly	120	(91.6)	68	(90.7)	52	(92.9)	0.758	107	(96.4)	62	(98.4)	45	(93.8)	0.314
(8) Do not place small objects	125	(95.4)	69	(92.0)	56	(100.0)	0.037	107	(97.3)	61	(96.8)	46	(97.9)	1.000
(9) Keep something hot away from the baby	117	(89.3)	68	(90.7)	49	(87.5)	0.580	108	(97.3)	60	(95.2)	48	(100.0)	0.257
	mean	(SD)	mean	(SD)	mean	(SD)	*p* ^d^	mean	(SD)	mean	(SD)	mean	(SD)	*p* ^d^
Knowledge of injury prevention (mean (SD))														
(1) Use a firm mattress: 4 queries	1.72	(1.5)	1.67	(1.5)	1.79	(1.5)	0.670	3.19	(1.11)	3.40	(0.91)	2.92	(1.29)	0.055
(2) Keep soft objects away from the baby’s head: 4 queries	2.64	(1.5)	2.64	(1.6)	2.64	(1.4)	0.816	3.61	(0.86)	3.76	(0.64)	3.42	(1.05)	0.032
(3) Make sure that there is no space: 3 queries	0.82	(0.8)	0.73	(0.8)	0.93	(0.8)	0.150	1.74	(0.94)	2.02	(0.89)	1.38	(0.89)	<0.001
(4) Do not use bed guard: 3 queries	0.14	(0.4)	0.15	(0.4)	0.13	(0.4)	0.664	0.60	(0.83)	0.92	(0.92)	0.19	(0.45)	<0.001
(5) Do not place your baby on high surfaces: 3 queries	1.59	(0.9)	1.53	(1.0)	1.66	(0.9)	0.464	2.07	(0.92)	2.19	(0.93)	1.92	(0.90)	0.096
(6) Keep crib sides raised: 2 queries	0.80	(0.8)	0.88	(0.8)	0.70	(0.8)	0.153	1.19	(0.72)	1.27	(0.68)	1.08	(0.77)	0.206
(7) Use the baby carrier correctly: 4 queries	2.24	(1.2)	2.21	(1.2)	2.27	(1.2)	0.770	3.26	(0.99)	3.40	(0.96)	3.08	(1.01)	0.036
(8) Do not place small objects: 4 queries	2.28	(0.9)	2.16	(1.0)	2.45	(0.8)	0.129	2.83	(0.87)	3.03	(0.82)	2.56	(0.87)	0.005
(9) Keep something hot away from the baby: 3 queries	1.09	(0.7)	1.03	(0.6)	1.18	(0.7)	0.182	1.55	(0.72)	1.67	(0.70)	1.40	(0.74)	0.051

^c^ Fisher’s exact test. ^d^ Mann–Whitney U test. Abbreviations: SD, Standard Deviation.

## Data Availability

The data presented in this study are not publicly available because of privacy restrictions.
